# Editorial: Dietary patterns, biomarkers, and health outcomes: an epidemiological perspective on the Mediterranean diet, sleep, and chronic disease

**DOI:** 10.3389/fnut.2026.1999927

**Published:** 2026-10-29

**Authors:** Gizem Özata Uyar, Betül Kocaadam Bozkurt, Melahat Sedanur Macit Çelebi

**Affiliations:** 1 Department of Nutrition and Dietetics, Faculty of Health Sciences, Kırıkkale University, Kırıkkale, Türkiye; 2 Department of Nutrition and Dietetics, Faculty of Health Sciences, Erzurum Technical University, Erzurum, Türkiye; 3 Department of Nutrition and Dietetics, Faculty of Health Sciences, Samsun Ondokuz Mayıs University, Samsun, Türkiye

**Keywords:** biomarkers, chronic disease, dietary patterns, Mediterranean diet, nutritional epidemiology, sleep quality

When we opened this Research Topic, our starting premise was simple: diet rarely changes in isolation. Differences in eating habits often coexist with differences in sleep, physical activity, psychological wellbeing, and metabolic health. The Mediterranean diet provides a natural framework for exploring these relationships, not only because a substantial body of evidence links Mediterranean diet adherence with favorable health outcomes across major chronic diseases (1–3), but also because it represents a dietary pattern rather than the effect of a single nutrient. Our aim was therefore to bring together epidemiological studies that consider diet within a broader behavioral and biological network and extend this perspective to populations and health contexts that nutritional epidemiology does not always reach.

Taken together, the ten contributions largely fulfilled these objectives while revealing several recurring themes. One was the importance of the factors that shape dietary behavior itself. Öngün Yilmaz et al. found that greater nutrition knowledge was associated with healthier and more sustainable eating behaviors and closer adherence to the Mediterranean diet. Başar Gökcen et al. extended this perspective to metabolic disease: among individuals with metabolic dysfunction-associated fatty liver disease, uncontrolled eating was associated with serum alanine aminotransferase levels through a significant serial indirect pathway involving Mediterranean diet adherence and BMI. Mindful eating showed related associations, although the corresponding moderated mediation pathway was not statistically significant. Together, these findings illustrate how nutrition knowledge, eating behavior, diet quality, adiposity, and metabolic health may be interconnected.

A second and particularly consistent theme was sleep. Babtain et al. compared clients attending diet centers in Jeddah and observed the most favorable sleep profiles among those following a Mediterranean dietary program and the poorest among those following a low-calorie ketogenic diet; physical activity was also associated with sleep quality within the same sample. These findings are consistent with previous observational evidence linking Mediterranean diet adherence with better sleep quality (4), although the cross-sectional design does not allow the direction of these associations to be determined. Wang Q. et al. focused on a more specific dietary behavior—how frequently individuals added salt at the table—and found that frequent table salt use was associated with unhealthy sleep patterns. Depressive symptoms accounted for part of this association, and the findings were further examined in an external clinical replication sample. Together, these studies suggest that sleep may deserve greater attention alongside diet when considering lifestyle-related health behaviors.

The psychological dimension extends this picture further. Boaz et al. examined Mediterranean diet quality and anxiety during armed conflict and identified an important nuance. The Mediterranean diet score itself was not associated with moderate-to-severe anxiety, yet participants with anxiety were considerably more likely to report deterioration in specific eating behaviors, including lower fruit and vegetable consumption, greater snacking, weight gain, and food insecurity. This distinction is informative: a summary dietary score may remain relatively stable even when meaningful changes are occurring in the individual behaviors it represents. In Wang J. et al.'s analysis of the English Longitudinal Study of Aging, depressive symptom severity partially mediated the association of poor and short sleep with incident osteoarthritis. Although mediation analysis cannot establish causal or temporal ordering, these findings suggest that depressive symptoms may represent an important intermediate pathway linking sleep characteristics with osteoarthritis risk.

The interplay between nutrition and sleep was particularly apparent in studies of aging. Zhang et al. found that higher intakes of vitamins B6, B12, and folate were associated with lower odds of cognitive impairment among older adults, whereas poor sleep quality was associated with higher odds. Of particular interest was the evidence of additive interaction: inadequate B-vitamin intake combined with poor sleep was associated with greater odds of cognitive impairment than either factor alone. These findings raise the possibility that nutrition and sleep may be more informative when considered together rather than as isolated determinants of cognitive health. Xiao et al. approached chronic disease risk from a biomarker perspective, reporting that a composite measure combining the triglyceride-glucose index and a body shape index was associated with atherosclerotic cardiovascular disease. Inflammatory and oxidative-stress indicators were also implicated in this association, while healthy dietary patterns—including the alternate Mediterranean diet, HEI-2020, and DASH—appeared to modify it.

Two contributions broadened the scope of the Research Topic beyond its predominantly cardiometabolic focus. Zheng et al. examined coffee and tea consumption in the UK Biobank and reported non-linear associations with incident lung cancer, with moderate coffee consumption showing the most favorable association. However, heterogeneity according to smoking status and reliance on self-reported baseline beverage intake warrant cautious interpretation of these findings. Huang et al. examined dietary factors among occupational radiation workers in Guangzhou and found that higher vegetable, fruit, and tea consumption was associated with lower morbidity, whereas a stated preference for meat over vegetables was associated with higher morbidity. Occupational populations remain relatively underrepresented in nutritional epidemiology, and these findings highlight the value of incorporating dietary assessment into studies of occupational health.

The limitations of this Research Topic largely reflect broader challenges in nutritional epidemiology. Most of the included studies are cross-sectional, many rely on self-reported assessments of diet and sleep, and several use mediation models that can characterize statistical pathways without establishing their temporal or causal sequence. These mediation findings should therefore be interpreted as hypothesis-generating rather than as evidence of established causal mechanisms. Moving the field forward will require more than additional cross-sectional evidence. Prospective and interventional studies, repeated dietary assessments, objective measures of sleep such as actigraphy or polysomnography, harmonized inflammatory and metabolic biomarker panels, and greater representation of under-studied geographic and socioeconomic populations would strengthen the evidence base. The observed additive interaction between diet and sleep also raises a question that future intervention studies could address directly: whether strategies targeting both diet and sleep provide greater benefits than interventions focused on either factor alone.

The shared message of these contributions is that diet does not operate in isolation. Diet, sleep, psychological wellbeing, adiposity, and biological processes coexist within real populations, and considering them together may reveal relationships that would otherwise remain obscured. This Research Topic therefore supports a broader view of nutritional epidemiology—one in which dietary patterns are understood within the behavioral, psychological, and metabolic contexts in which they occur.
